# Modification of the Cavity of Plastic Injection Molds: A Brief Review of Materials and Influence on the Cooling Rates

**DOI:** 10.3390/ma14237249

**Published:** 2021-11-27

**Authors:** Maria C. Carrupt, Ana P. Piedade

**Affiliations:** CEMMPRE, Department of Mechanical Engineering, University of Coimbra, 3030-788 Coimbra, Portugal; m.cristina_borges@hotmail.com

**Keywords:** injection molding of plastics, molding cavity, metallic and ceramic coatings, cooling rate

## Abstract

In the 21st century, a great percentage of the plastic industry production is associated with both injection molding and extrusion processes. Manufactured plastic components/parts are used in several industry sectors, where the automotive and aeronautic stand out. In the injection process cycle, the cooling step represents 60% to 80% of the total injection process time, and it is used to estimate the production capabilities and costs. Therefore, efforts have been focused on obtaining more efficient cooling systems, seeking the best relationship between the shape, the quantity, and the distribution of the cooling channels into the injection molds. Concomitantly, the surface coating of the mold cavity also assumes great importance as it can provide increased hardness and a more straightforward demolding process. These aspects contribute to the decrease of rejected parts due to surface defects. However, the effect of the coated cavity on the heat transfer and, consequently, on the time of the injection cycle is not often addressed. This paper reviews the effects of the materials and surface coatings of molds cavity on the filling and cooling of the injection molding cycle. It shows how the design of cooling channels affects the cooling rates and warpage for molded parts. It also addresses how the surface coating influence the mold filling patterns and mold cooling. This review shows, more specifically, the influence of the coating process on the cooling step of the injection cycle and, consequently, in the productivity of the process.

## 1. Introduction

At the beginning of the 21st century, both injection molding (IM) and extrusion processes were responsible for absorbing 36% (in weight) of all plastic produced worldwide [1]. 

Since the 1980s in the previous century, micro-process fabrication began to emerge with the miniaturization of equipment and devices [2]. The IM process was not able to meet the market demand due to the size of the equipment. Thus, the micro-injection molding (μIM) process appears to be the most efficient process for mass production of micro plastic parts with the best cost/benefit [3]. However, this is not a reduced version of the classical IM process and requires new approaches in several areas, such as mold construction technology, raw materials, precision technology, and nanorheology. [2]. Some advantages of the μIM process include short processing time, suitability for large-scale production, molding, no need for post-processing and the capability to produce complicated 3D shape parts [3].

In the 1990s, plastic production increased along with the concerns about the environment and restrictions on CO_2_ emissions, resulting in the search for lighter materials to reduce the weight of vehicles. In this context, the microcellular materials developed between 1981 and 1984 and patented in 1984 became the most attractive alternative. In these materials, a heterogeneous nucleation mechanism is favored due to additives such as CO_2_, among others, although the primary function of this compound is as a blowing agent. The gas dissolves in the amorphous phase, causing the disorder of the crystalline part of the polymer. The absorbed gas forms spherical cells, reducing the weight of the material [4]. In 1998, the first machine for microcellular injection molding was built. Microcellular injection molding entails injecting a supercritical gas (usually carbon dioxide or nitrogen) into a molten polymer and allowing it to expand and fill the cavity, removing part of the previously injected polymer and forming a cellular structure. This approach is considered one of the most effective methods for reducing weight and cycle time in plastic components, especially those with thick walls [4,5]. These components present several problems, such as sink-marks or internal voids, that need long processing times to avoid them. The use of microcellular injection molding allows obtaining components with the absence of sink-marks and a lower warp in shorter injection cycles [5].

However, regardless of the type of injection molding process, the cooling step represents from 60% to 80% of the total injection process time [6,7], and it is used to estimate the production capabilities and costs [8]. Therefore, as the cooling step is shortened, a gain in productivity and a decrease in costs are observed. Consequently, efforts have been focused on obtaining more efficient cooling systems, seeking the best relationship between the shape, the quantity, and the distribution of the cooling channels into the injection molds [9]. This problem can be approached through different perspectives that can overlap the fields of new technologies, mold design, mold fabrication techniques, materials used in the fabrication of molds, and coatings of the mold cavity.

When heat transfer problems are considered, most of the literature is focused on solutions that may provide a very fast reading of the actual temperature in the mold cavity. Without accurate values in real-time of the mold temperature, the efforts to decrease the cooling stage of the injection cycle are seriously jeopardized. Ageyeva et al. [10] give a recent review of this approach that covers the established process monitoring techniques, such as direct mold cavity temperature, with standard sensors and the newly developed sensors. The review is related to Industry 4.0 that demands a great deal of data for manufacturing process control in plastic production.

In what concerns new technologies developed, one of the most interesting ones in this field is called rapid heat and cooling molding (RHCM) [11]. This technology claims full temperature control, i.e., the required temperature in the cavity is under control at any injection stage. An experimental study performed by Sanchez et al. [12] using RHCM to optimize injected parts with amorphous polymers showed the advantages of the technique. However, the authors also observed an important cycle time increase when compared with conventional injection molding.

Recent technological advances in cooling system design, which have accompanied the exponential growth of additive manufacturing processes (AM), are primarily related to the production of dynamic conformational cooling channels in AM or hybrid manufacturing systems (AM and conventional producing technologies) [13,14,15,16].

From another point of view, the use of coatings on the cavity surfaces of injection molds has become a practical resource for improving the plastic injection processes [17]. Therefore, thin films deposited by physical vapor deposition (PVD) and chemical vapor deposition (CVD) techniques are mainly used in the plastic injection mold industry to increase corrosion resistance, wettability, improve the hardness and tribological properties of the molding cavities, and increase the lifetime of the mold [18]. The coatings can also be used as a thermal barrier to decrease the damage due to high thermal stress [19]. The metallic films have been used almost exclusively as interlayers to improve the adhesion of the ceramic coating to the metallic injection mold [20]. The improvement of deposition techniques leads to the development of multilayer coatings, which can combine different materials in functional gradient, increasing the possibilities of combining properties [21].

This manuscript focuses on the materials and deposition techniques used in the molding cavity and the results of the influence of cooling channels design and surface coating on the filling and cooling cycles of the injection cycle. The approach presented in this brief review is often disregarded in the published literature on this subject.

## 2. Injection Molding Process

This section intends to briefly describe the process and highlight some of the problems and solutions attempted to overcome them. The injection molding process is the primary process used for manufacturing plastic parts on a large industrial scale, where the molten polymer is injected under pressure into a mold. Due to its efficiency in producing pieces of varied sizes and shapes, with excellent accuracy and high productivity, this process has a reasonable cost/benefit ratio [22].

Although the basic concept of functioning had remained practically the same since 1951, when Willert developed the modern reciprocating-screw machine, it is a continually evolving process aimed at increasing the melting rates and better temperature control. Thus, it has been widely used since the 1950s [22]. The reciprocating screw is also known as the single-stage injection molding machine (IMM), and together with the two-stage IMM (piggy-back IMM), it is the most popular IMM. In addition, there are IMMs with three or more stages [1]. The injection molding machine comprises three main parts: the clamping unit, the injection unit, and the power unit [1,22].

The power unit supplies the energy (hydraulic and electrical) necessary for all IMM movements during the injection process [22]. The injection unit, also called the plasticator [1], consists of the hopper and the heating cylinder. The raw plastic pellets are stored in the hopper before introducing them into the heating cylinder [17]. The heating cylinder, or barrel, is responsible for heating and melting the polymer. Along the length of the barrel, there are three distinct heating regions: rear, center, and front. The heating of each region is individually controlled, and an electrical heater band does it (three heater bands per zone approximately), positioned on the outside of the barrel and minimally separated from each other.

The heater bands are electrically driven [17]. Thus, the raw polymer pellets are gradually melted as the screw transports them through the heating cylinder. The clamping unit is driven automatically, closing and opening the mold at each injection cycle, releasing the finished part [22]. The force applied to the unit during the filling step must support the pressure inside the mold cavity to keep it closed. The clamping force depends on the type of polymer being injected [1].

Injection molding is a cyclical process that can be divided into four steps: (1) Melting of raw polymer pellets; (2) Injection of molten material into the mold cavity (filling); (3) Cooling of the mold (solidification); and (4) Ejection of the workpiece from the mold [23,24].

Although the injection molding process can appear simple, it is a dynamic and nonlinear process in which the cycles are thermodynamically complicated because of the interaction between machine parameters, material properties, and process variables [22]. Due to this, the polymers undergo thermodynamical, rheological, thermal, and mechanical changes [25].

During all injection processes, some variables need to be controlled to ensure the process efficiency and quality of parts. The main variables are, in decreasing order of importance: temperature (of melt, of mold, of the hydraulic system, and of ambient), pressure (of injection unit and clamp unit), time (gate-to-gate and gate close, mold close, initial and hold injection, cooling, screw return, mold open, and ejection), and distance (mold close, injection and injection-hold, cushion, screw return, mold open, and ejection) [17].

The mold temperature and the cavity pressure greatly influence both parts’ quality and properties during the injection process [22]. These variables are integrated, and one influences the other. The injection time can be minimized if the filling temperature is kept higher than the melt temperature. On the other hand, a lower filling temperature drives higher cavity pressure [23].

## 3. Heat Transfer during Injection Molding

The cooling cycle (including the solidification time) has received the special attention of researchers in recent decades. Due to the low thermal diffusivity of polymers [23] it represents 60% to 80% of the total injection process time [6,7]. The less time the cooling cycle lasts, the faster the next step starts. Consequently, the process becomes more efficient. Thus, cooling is the parameter used to estimate the production capabilities and costs [8]. Besides, the cooling also influences the quality and mechanical properties of workpieces. The residual thermal stress and warpage are reduced by uniform cooling [26]. Depending on the cooling system, at least 60% of the surface and apparent defects [27].

Optimizing the cooling cycle (packing + solidification) is mandatory to reduce the heat transfer rates and keep uniform parts during cooling. However, the heat transfer rate between the polymer and mold does not remain constant during the injection molding cycle and influences the contraction of both workpiece and mold during the solidification, the filling pressure, and the cooling performance. It depends on the following [27,28]:

Polymer characteristics—amorphous materials can cool faster than semi-crystalline ones, as the latter need a slower cooling rate to allow the structural order of the macromolecules. The cooling rate should allow for the thermal phenomenon of cooling crystallization to occur [29,30].

Process parameters—the pressure inside the mold cavity and the temperature of the mold and melted polymer.

Mold—design of the cavity, surface roughness, design of the cooling system.

In recent decades, researchers have been working to develop a more efficient cooling system. The oldest article found about the improvement of the cooler system in injection molding dates from 1969 [31], and probably it is the first. Thenceforth, it is unanimous among researchers that to improve the efficiency of cooling systems, it is necessary to find the best relationship between the shape, the quantity, and the distribution of the cooling channels into the injection molds [9].

The most common cooling channel is made by traditional machines, such as CNC drilling or electrical discharge [9,32]. However, these manufacturing techniques limit the channel design to round profiles in straight lines [33], limiting the number and placement of channels inside the mold cavity [34,35]. Consequently, heat transfer through the mold is not homogeneous, resulting in different shrinkage that drives to warpage and increased cooling time [33] due to the heat transfer’s influence on the kinetics, crystallinity degree, and microstructure of polymers [36].

**Figure 1:**
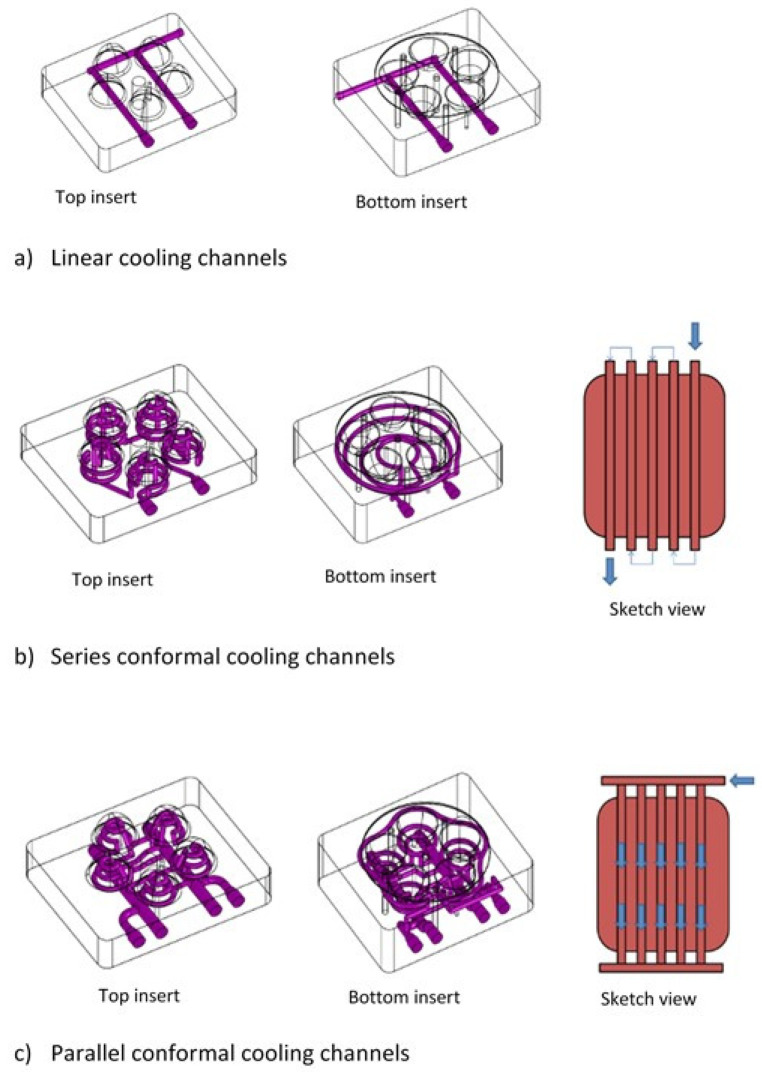
Schematic representation of cooling system types [33]. Open access, Copyright: Associação Brasileira de Polímeros.

Over the past ten years, researchers have been using 3D modeling software and additive manufacturing processes to develop cooling channels that follow the cavity geometry, known as conformal channels. Figure 1 shows a schematic representation of a linear cooling system (conventional) and a conformal cooling system [33].

The results observed by several researchers in the last decade regarding the cooling channel design influence on the heat transfer rate between the mold and the polymer are listed in Table 1. 

Conformal cooling channels force the coolant fluid to pass very close to the mold surface, promoting homogeneous heat transfer during cooling, reducing cooling cycle time, and thus lowering operation costs [33].

In addition, to influencing the cooling speed, the design of the cooling system influences the level of warping of the produced parts. Huang et al. [43] used a Taguchi robust design process to evaluate the best parameters of temperature distribution and the level of warpage of the parts during the injection process, having as control variables injection time, material temperature, mold temperature, injection pressure, packing pressure, packing time, cooling liquid, and cooling temperature. The ideal temperature and warpage parameters determined (warpage of 0.783 mm and an average temperature of 235.2 °C) were then used to explore three different cooling system designs: original cooling, square cooling, and conformal cooling.

**Figure 2:**
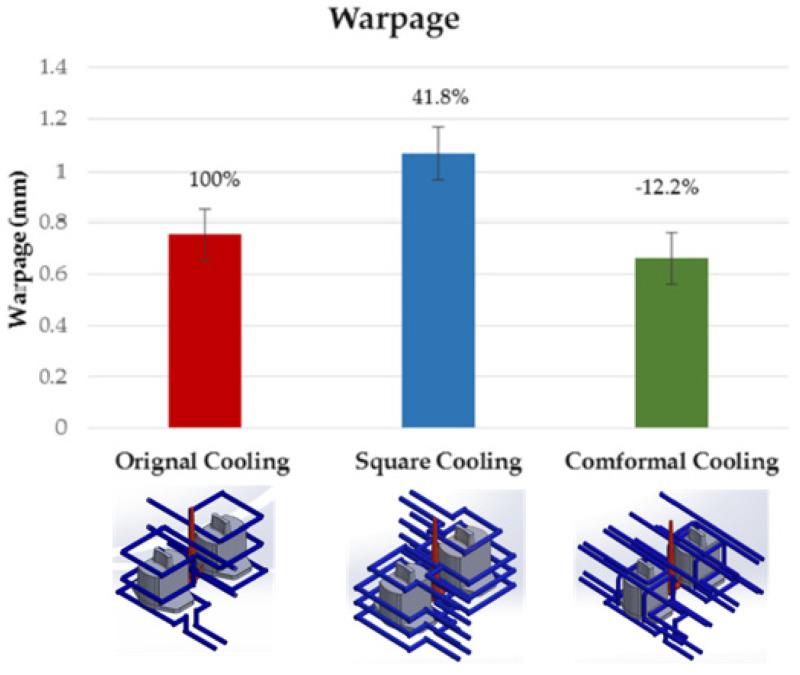
Comparison of warpage deformation values for different cooling circuit systems [43]. Open access, Copyright: MDPI.

The conformal cooling design has a better warpage value (0.661 mm), and the original channel has the worst (1.068 mm). Figure 2 shows a comparison between the three designs. In the original design, square cooling showed an increase of 4.8% in warpage, while in conformal cooling, the warpage is reduced by 12.2%.

Moreover, besides the spatial distribution of the conformal cooling channels near the mold cavity, the shape of their cross-section also influences the thermal efficiency of the cooling system. Le Bot et al. [42] showed that under the same cooling conditions (cross-sectional area and coolant flow rate), the time necessary to complete solidification of parts could be about 3% lower when the cooling channels present a rectangular section instead of a cylindrical one.

**Figure 3:**
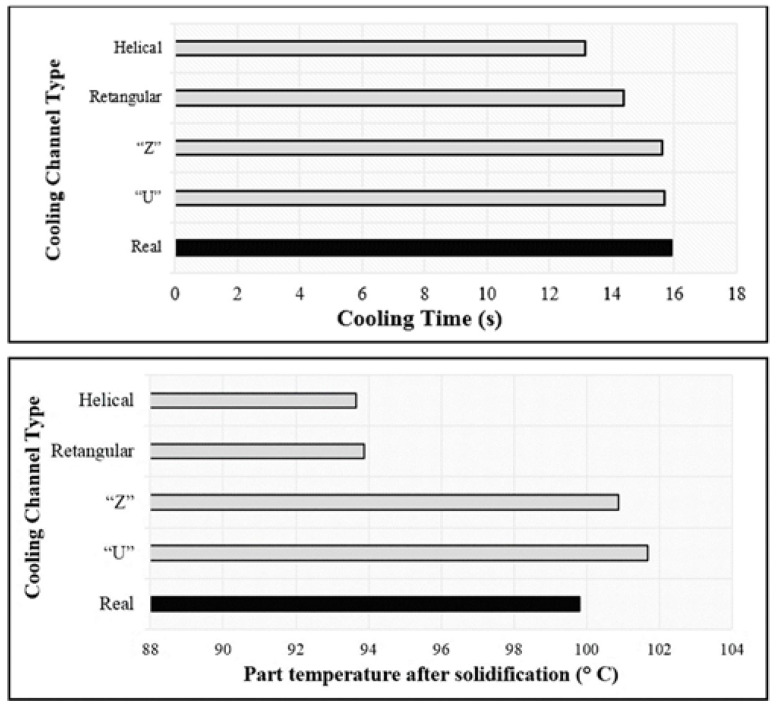
Comparison of the performance of different cooling systems geometries [44]. Open access, Copyright: Associação Brasileira de Polímeros.

In 2020, Gruber and Miranda [44] compared the efficiency of different cooling systems designs used in plastic injection molds (helical, rectangular, “Z”, ”U” and, real), and the impact of each of them on the process cost, using commercial software simulation. Figure 3 shows comparison graphs of the efficiency of the evaluated cooling systems. In Table 2, a comparison of the productivity of each cooling system is given.

From the obtained results, it is clear the influence of the cooling system design on productivity. The helical geometry is the best choice because it allows an increase in the number of produced parts. This increase is due to the decrease of both the cooling time and the temperature part after solidification. These two factors contribute significantly to the decrease of the cooling step and, consequently, decrease the time of each injection cycle.

It must also be briefly mentioned that some researchers are now exploiting the constructal theory [45] in the attempts to decrease the cooling time. This theory, first developed in 1996, describes the generation of flow systems configuration considering structure, shape, and architecture. The idea is based on Nature, considering, for example, the type of networks inside cactii that allow them to bear high temperatures and, simultaneously, act as reservoirs of water. The freedom of design given by constructal theory is another factor that contributes to the optimization of the design of the flow configurations [21,39].

Besides obtaining the best conformal cooling design, it is also essential to find a way to apply the manufacturing technique to as many cavity shapes as possible [32,42]. The mold cavity with the conformal channel can be made by additive manufacture as selective laser melting (SLM) and direct metal laser sintering (DMLS) [33].

Additive manufacturing techniques are still very expensive, and optimized flow designs based on constructal theory are far from an industrial reality. It is also important to improve the mold cavity design to reduce the raw material quantity, maintaining the mechanical resistance [34].

In the last decade, very few solutions to improve the cooling rate in the plastic injection process have been patented. In addition to modifications to the cooling channels, the designs also feature a coolant option. Table 3 lists the patented designs with a brief description.

Hendry [48] proposes heating the mold cavity surface using sprayed steam. When the cavity reaches the desired temperature, steam is condensed and removed from the mold cavity so that the molten polymer is then injected. Consequently, it reduces the temperature difference between the mold and the molten polymer by reducing the heat exchange rate between the two. However, this solution does not reduce the injection cycle time.

Neufarth et al. [47] proposed a cooling system that combines the passage of a refrigerant fluid with steam through a system of refrigeration channels in a simple configuration. The fluid is sprayed into the cooling channels, and as it evaporates, it exchanges heat more efficiently with the mold, reducing the cooling time. The system had a condenser unit to remove steam and refrigerant, called exotic, which has a thermal conductivity of around 1 W/mK or better. 

Altonen et al. [46] present a proposal to improve thermal efficiency in the injection process for the same cooling channel system used by Neufarth [47]. The researchers replaced the material with high hardness and high thermal conductivity of the plate where the cooling channels are located by a material with excellent thermal conductivity, using water as the coolant. The plate with a simple system of cooling channels is in direct contact with the plate where the mold cavity is located. Through simulation, as the plate with the channel systems has better thermal conductivity, heat extraction is facilitated, and thermal efficiency improves.

## 4. Coating of the Injection Mold Cavity 

The use of thin films to coat plastic injection mold cavities aims to increase corrosion resistance and improve tribological properties, specifically reducing the coefficient of friction [18]. In addition, they can also be used as a thermal barrier to decrease the damage of the mold cavity due to high thermal stress, mainly in micro/nano injection molding processes. Thermal stress can develop at the interface between the mold and the injected material, compromising repeatability and the surface quality of final parts [19]. According to the published research, ceramic thin films are the most used for this purpose.

The use of ceramic coatings can be tuned according to their chemical nature/composition. Among the several types of coatings, nitrides gain prominence in the literature. Titanium-based nitrides are applied mainly to improve tribological properties [49,50,51], while zirconium-based nitrides act as thermal barriers to reduce the cooling rate, mainly in microinjection [19,52]. However, ternary metal and nitride alloys (e.g., TiSiN and TiAlN) have gained ground and replaced binary alloys in many applications. The addition of silicon improves the thermal, mechanical, and chemical properties and makes it one of the best metal nitride materials [21].

Coatings used to enhance corrosion resistance are based on transition metal nitrides, mainly chrome-based nitrides (Cr-N) [53,54]. With the appropriate addition of Ni, in the range of 20–40 at.%, the fracture toughness and wear resistance could be improved compared to the Cr–N binary coating while maintaining a high hardness value [55]. TiAlN coatings also provide good corrosion resistance because, at high temperatures, the aluminum tends to form an Al_2_O_3_ layer on the surface in a passivation process [19]. However, coatings deposited by PVD techniques can sometimes present defects, such as pores and pinholes, which compromise the ability of the coating to protect the metal substrate from corrosion, especially in more aggressive environments [51,54].

In recent decades, diamond-like carbon (DLC) and hydrogenated amorphous carbon (a: C-H) coatings have been used to improve the lubrication properties of [56,57,58]. In this case, the coating is usually deposited either by CVD or PVD to improve the wettability and filling flow. On the other hand, the adhesion and the injection force are reduced. These variables influence the replication and the quality of the surface of the injected parts. They have been evaluated for different ceramic coatings and their interaction with different melted polymers [57] and glass fiber-reinforced plastics [18,59] during the injection process. 

**Figure 4:**
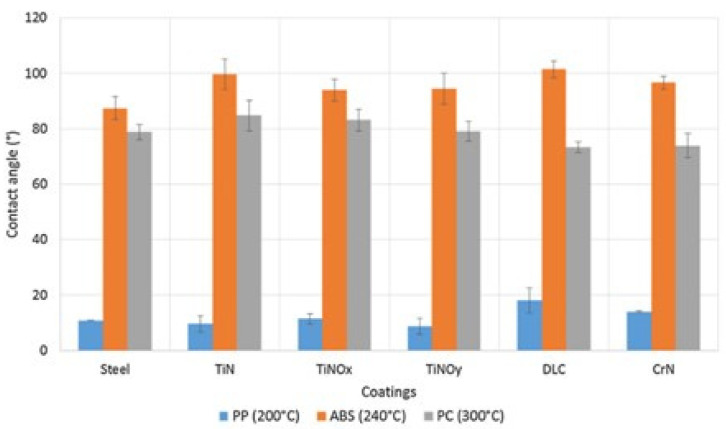
The contact angle of the PP, ABS, and PC melt on the different surfaces at a temperature of 200 °C, 240 °C, and 300 °C, respectively [60]. Reproduced with permission from Elsevier.

The wettability of the coating also depends on the interaction with the molten polymer. Vera et al. [60] evaluated the wettability of coated and uncoated steel surfaces, using three types of polymers (polypropylene (PP), acrylonitrile-butadiene-styrene (ABS), and polycarbonate (PC)), by measuring the contact angles at the melting temperatures of each polymer. Figure 4 presents the results of this comparison.

Taking the uncoated surface (steel) as a reference, all tested coatings (TiN, TiNO_x_, TiNO_y_, DLC, and CrN) increased the average contact angle between the ABS and the surface, indicating a decrease in wettability. For PC, only TiN and TiNOx coatings promoted a small increase in the contact angle, while DLC and CrN reduced the contact angle to values below that of the uncoated surface. Concerning PP, only DLC and CrN coatings did not prove to be efficient in improving wettability. Therefore, due to a more difficult wettability, only DLC can be applied as a lubricant in molds for PP and ABS injection. If only ABS injection is considered, both DLC and TiN proved to have a better lubricating action. 

However, ceramics are not the best choice from the thermal efficiency point of view during the cooling stage. Ionic and covalent bonds chemically bond atoms in this class of materials. Consequently, there are no free electrons, implying that these materials have low electrical conductivity, low thermal expansion coefficient, extremely high hardness, and low toughness [20,61]. On the other hand, metallic materials are ductile, have some toughness, good electrical and thermal conductivity because they are formed by metallic chemical bonds with the intense movement of free electrons [61].

These differences, especially when considering the thermal properties between the ceramic coating and the metallic substrate, are a limiting factor since the coating is not able to accompany the elastic behavior of the mold, which occurs due to the temperature variation during the injection process, resulting in the cracking and peeling of the coating [62]. Good adhesion of the coating guarantees its durability and a longer service life [20]. Enhancing the ceramic coatings’ adhesion to the metallic substrates has been the main focus of many researchers over the last years. 

The adhesion of the coating to the substrate can be explained from a thermodynamic point of view as a change in the surface energy when the interface is formed [20]. The adhesion force depends on the chemical and physical interactions between the coating and the substrate [19]. Therefore, to improve the interface adhesion, three points must be carefully considered: low surface energy, solid and stable chemical bonds between coating and substrate, and a low-stress gradient in the interface [19]. 

The surface free energy of both the coating and injected material surfaces plays an important role in the mold-release capability and non-sticking condition. Injection molds with lower values of free surface energy are more suitable as non-sticky surfaces [20]. The non-stick condition is associated with the work required to separate the melted polymer from the mold, known as adhesion work [63], where the better non-stick condition is associated with low adhesion work. For a strong and stable chemical bond between the coating and the substrate, the most common strategy reported by researchers is the use of intermediate layers, with chemical compositions that resemble both the substrate and the nitride coating. These layers usually present a metallic character and can be constituted by a single element or a combination. The hardness of the interlayer should be close to the hardness of the material of the mold [20]. Additionally, a low-stress gradient in the interface is necessary because if two materials present high interfacial tension, there will be no adhesion [64].

**Figure 5:**
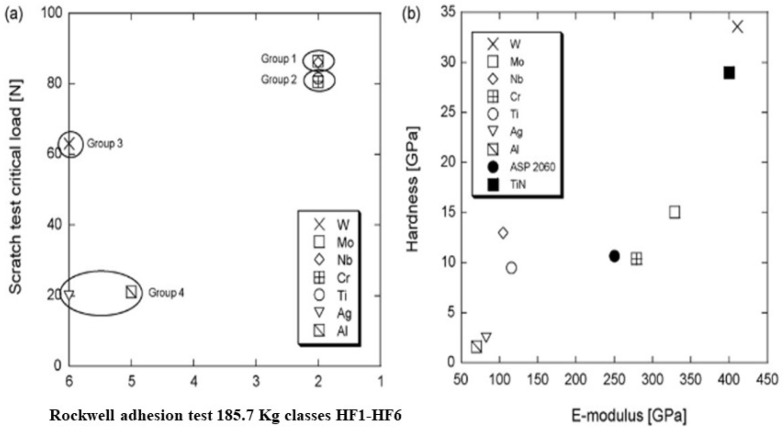
(**a**) Compilation of the results from the adhesion test for each sample. The y-axis is the mean value of the critical loads in scratch testing. (**b**) E-modulus vs. hardness is shown for each sample plus the ASP 2060 substrate and TiN coating [19]. Reproduced with permission from Elsevier.

Gerth [19] evaluated the efficiency of intermediate metallic layers with thicknesses within the range 100–150 nm, constituted by metallic elements (W, Mo, Nb, Cr, Ti, Ag, Al) from different groups of the periodic table, in the adhesion of the ceramic coating (TiN) within 3.1 ± 0.5 μm of thickness on a metallic substrate. The samples were characterized by scratch and Rockwell tests. Figure 5 shows the results of these tests. 

The intermediate layers constituted by group 1 (Nb, Mo), and group 2 (Ti, Cr) showed the best adhesion results in both tests. The interlayer of group 3 (W) showed very low resistance to detachment when submitted to the Rockwell adhesion test. The elements of group 4 (Al, Ag) show extremely poor adhesion in both tests. These results corroborate the concept that an intermediate layer with a hardness close to the coating layer promotes better adhesion of the ceramic coating to the metallic substrate.

The main application of thin films in plastic injection molds is to improve wear resistance and reduce the flow resistance of the molten polymer and the adhesion strength. Consequently, most of the applied coatings are ceramic. With the rise of the microinjection molding process in the last decade, these coatings are also used as a thermal buffer to homogenize the temperature distribution throughout the mold cavity [52,64]. 

In the microinjection molding process, higher injection pressure and velocity are required to prevent premature solidification of the material [61]. The conjugation of these parameters increases the friction between the mold cavity and the molten polymer, which is also responsible for structural damage due to the high thermal stress caused by the heterogeneous temperature distribution [19]. 

In the last decade, very few articles have mentioned applying ceramic thin films as a thermal barrier. Table 4 lists the results found in the literature where ceramic coatings are used as a thermal buffer. The vast majority of articles emphasize the applications of these coatings as solid lubricants and as a hard coating, thus increasing wear resistance.

However, when these coatings act as a thermal barrier, there is a reduction in the heat exchange capacity between the mold and the molten polymer, resulting in better temperature distribution and reduction in the warping and distortion rate. These conditions are more favorable for the microinjection process. However, once again, the overall injection cycle time is not diminished as the cooling step increases its duration.

The DLC coatings deposited by CVD have been used as a thermal buffer in microinjection molds due to their high hardness, thermal conductivity, increased thermal shock capacity, and low friction coefficient [64].

**Figure 6:**
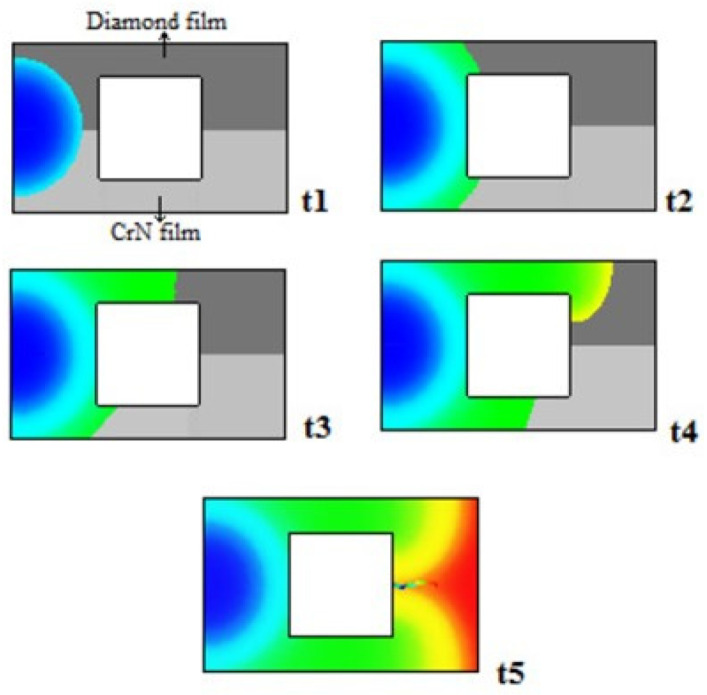
Influence of chemical composition of the coating on the plastic flow during filling. (t = time, with t1 < t2 < t3 < t4 < t5) [64]. Reproduced with permission from Elsevier.

Santos and Neto [64] analyzed the efficiency of this coating as a thermal buffer. Using computer simulation, they studied the temperature distribution during the filling phase of a cavity coated with DLC and compared it with another coated with CrN. Figure 6 shows the flow behavior during the filling stage.

The DLC film acted as thermal resistance, decreasing the strong influence of the polymer/mold interface heat transfer and balancing the temperature. The temperature is evenly distributed over the diamond coating as compared to the CrN coating. 

Thermo resistance can adjust, point to point, the heat transfer during the cooling according to demand on the surface of the cavity. Thermo resistance varies with thermal conductivity and the thickness of the coating. Since the thermal conductivity of the films can be altered by the chemical composition and thickness, the thermal resistance can also be adjusted [52].

**Figure 7:**
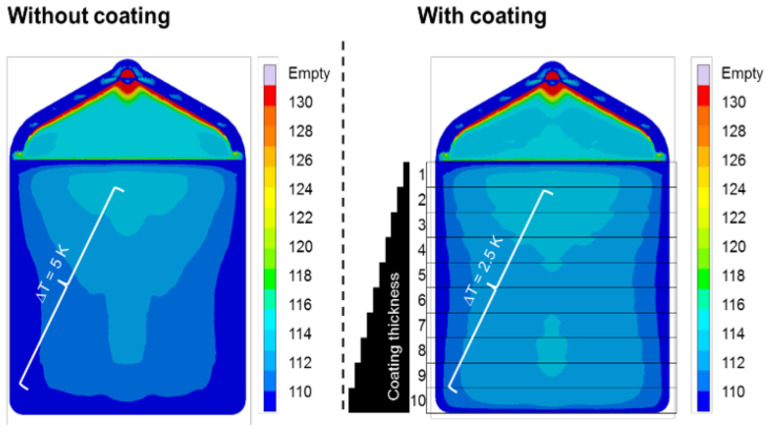
Simulated temperature distribution of a part after 10 s cooling [54]. Open access, Copyright: IOPScience.

Bobzin et al. [51] analyzed the influence of the coating of yttria-stabilized zirconia (YSZ) on the temperature distribution over a plate (Figure 7).

The image shows that the YSZ coating was efficient in making the temperature distribution more homogeneous. As a result, the intensity of the thermal gradient across the part was reduced by 50%.

**Figure 8:**
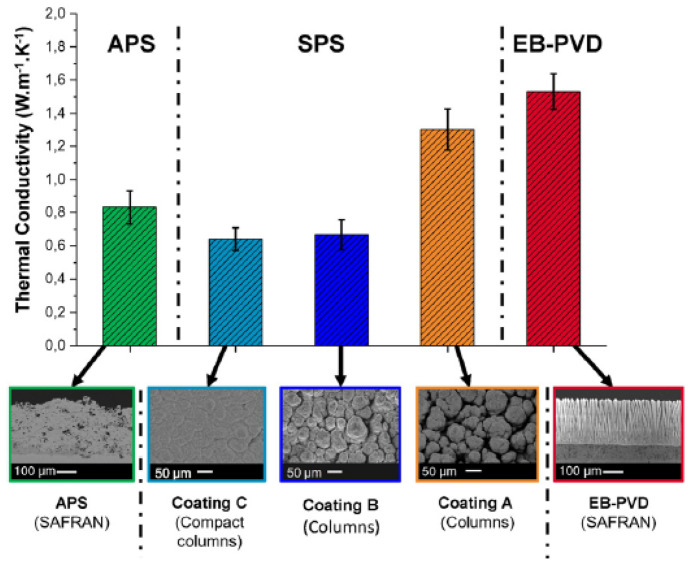
Thermal conductivities at 25 °C of YSZ samples performed by SPS compared to YSZ obtained by APS and EB-PVD [6]. Reproduced with permission from Elsevier.

The thermal conductivity of the YSZ coating can be associated with the morphology of the coating layer. Bernard et al. [65] evaluated the influence of the coating layer morphology on the thermal conductivity of YSZ, deposited on stainless steel. For this, it used three different deposition processes: suspension plasma spraying (SPS), electron beam physical vapor deposition (EB-PVD), and atmospheric plasma spraying (APS). Figure 8 shows the results.

The coating obtained by EB-PVD, with an open and parallel columnar structure, has the best thermal conductivity value. On the other hand, C coatings of compact morphology obtained by SPS have the worst thermal conductivity. According to Bernard [65], the gaps between the columns of the EB-PVD coating allow the passage of air, facilitating the exchange of heat. As these gaps do not exist in the compact coating (coating C) obtained by SPS, heat exchange is not facilitated. The other images in Figure 8 show that structures with a morphology that facilitates air passage have better thermal conductivity.

Therefore, for applying YSZ coatings as a thermal barrier, coating processes and parameters that lead to more compact coating layer morphologies must be chosen.

**Figure 9:**
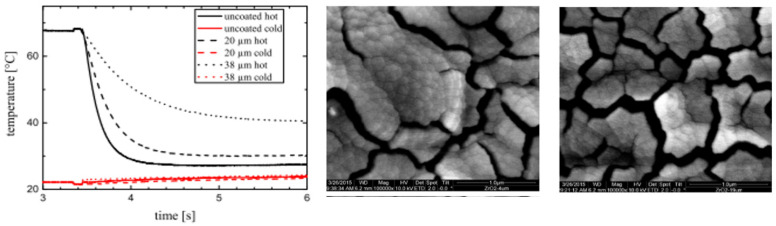
Time-dependent temperature measurements of two zirconia-coated and one uncoated sample, and the morphology of ZrO_2_ layers, with 4 μm (middle) and 20 μm (right) [18]. Reproduced with permission of John Wiley and Sons.

Atakan et al. [18] investigated the thermal barrier layer of zirconium dioxide (ZrO_2_), deposited by CVD, to avoid induction heating, reducing the heat transfer rate from the polymer to the cavity. In Figure 9, we can see (on the left) how the heat transfer rate varies depending on the thickness of the coating layer and how the thickness of the layer changes the structure of the thin film. There is a layer with 4 μm of thickness in the middle and, at right, a layer with 19 μm of thickness. The layer with 38 μm presented a lower heat transfer rate.

Some researchers [66] evaluated the electrical properties of thin coatings deposited via PVD to design sensors for temperature measurement. These coatings should facilitate an online measurement of the surface temperature and offer a thermal resistance of up to a few hundred degrees centigrade to allow its application in various technical applications. Hard coatings were selected because they are state-of-the-art as wear protection for numerous manufacturing technology tools. The coating layers (TiAlN and Al_2_O_3_ as an electrical insulator) were applied over a metallic substrate (X37CrMoV5-1). Over the Al_2_O_3_ layer, the sensor layers were applied, one pair formed by CrN/AlN and TiAlN and the other pair by CrAlN and one TiAlN. The suitability of both hard coatings as sensors for measuring temperature has been proven up to a temperature of T ≈ 250 °C. Comparing both sensor coatings, CrN/AlN + TiAlN is characterized by significantly better measurement accuracy, i.e., better response behavior and less measurement drift, compared to CrAlN + TiAlN. Hard nitride coatings have the potential to be used as thin-film thermocouples.

For the same applications, researchers studied the dependence of the effective thermal conductivity of the coating on its columnar structure [67]. The coating was deposited by electron beam physical vapor deposition (EB-PVD) to optimize the thermal insulation of the coating considering mechanical stress restrictions. A three-dimensional finite element method was used to predict the thermal contact resistance through the interfaces between the adjacent columnar structures. The results show that the thermal conductivity decreases with an increase in the angle and the diameter of the columns, leading to an increase in mechanical stress on the cladding root. Increasing the thickness of the cladding would increase thermal conductivity effectiveness and an increase in mechanical stress at the cladding root when cladding columns are tilted.

Although without specifying the application for the coating of cavity molds, new enamel coatings were synthesized with silicon nitride with the contents of 0, 1.5, 2.5, 3.5, 5, and 7.5 (wt.%) applied on the metallic substrate by spraying followed by sintering [68]. The samples were subjected, among other analyses, to thermal shock and potentiodynamic. Polarization tests were conducted to evaluate the microstructure and engineering properties of the enamel coatings. The results showed that the addition of silicon nitride, even leading to high internal porosity, increased the enamel corrosion resistance. Silicon nitride-modified enamel coatings have survived over 100 cycles of temperature shocks, allowing them to be applied in environments with high-temperature fluctuations. These results can provide a solution for protecting steel against exposure to high temperatures and corrosive environments.

These works, which use hard insulating ceramic coatings, do not evaluate their impact on the cooling rate of the injection cycle when used in the plastics injection molding process. Due to their thermal insulation properties, it is expected that these coatings will increase this step of the injection cycle. Consequently, they do not contribute to the increase in productivity, as mentioned before.

Usually, in injection molding processes, metallic films have been used as interlayers to improve the adhesion of the ceramic coating to the metallic substrate (injection mold cavity) [20]. However, none of the studies analyzes the performance of metallic thin films as heat transfer rate optimizers. No articles have been found where metallic thin films were used to optimize cooling rates and heat transfer during the cooling phase.

Xie et al. [69] analyzed the performance of thin metallic films (Al, Ti), aiming to increase the weld line’s performance in microinjection processes. The weld line is a common defect in microinjection molds that drive decreases in the mechanical and superficial properties of parts. 

The results obtained by Xie et al. show that the metallic thin films improved the mechanical properties of the weld line. Both reinforced the tensile strength, but the Al provided better results, leading to a 5–13% increment against 3% of Ti increment. Furthermore, the modulus of elasticity has also been improved by coatings [69]. The authors attribute this increase to releasing the stress concentration at the weld line area by transmitting the stress load to a larger area of the sample surface.

Oliveira et al. [70] developed a thermosensitive thin film to control the temperature during the injection molding process. Ti and Cu thin films were doped with N_2_ and O_2_, resulting in a TiCu(N,O) thin film. The thin film was deposited with different percentages of the reactive gases. 

**Figure 10:**
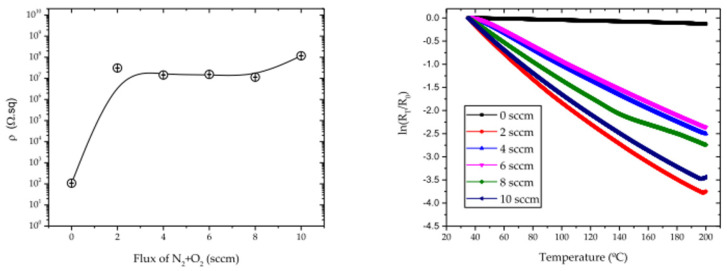
Influence of N_2_+O_2_ flux on the thermal properties of the coatings [70]. Open access, Copyright MDPI.

The N_2_+O_2_ percentage influenced the thermal properties of the deposited films. Figure 10 shows how (at left) the electrical resistivity varies with the relative content of N_2_+O_2_ at room temperature and how the thermoresistive response of each film (sputtered on glass substrates) varies with temperature.

Oliveira et al. [70] concluded that the temperature distribution in the mold, directly during the heating and cooling process, is possible with this thermoresistive sensor based on the TiCu(O,N) system.

Recently, an alternative to a more homogeneous temperature distribution during microinjection is to deposit electrically conductor coatings directly over the mold cavity. The Joule effect heats the coating to a temperature close to the temperature of the molten polymer. Ni-based alloys are the most used as thermal coatings, but semiconductor materials can also be used. Bobzin et al. [71] applied a three-layer coating, where the outer layers were Al_2_O_3_ (to increase wear and corrosion resistance) and the intermediate layer TiOx/Cr_2_O_3_ and obtained positive results regarding the temperature distribution during molding. The results were positive because the temperature distribution on the specimen surface was homogeneous, and no change was observed over 10,000 thermal cycles.

Polymeric coatings have also been applied to microinjection molds to reduce the cooling rate, distortions, shrinkage, and other defects. Kim and Song [2] applied, via spray coating, a polymeric polyimide (PI). The micro-injection coated mold was tested by molding a light guide plate (LGP) in polycarbonate (PC). The PI coating reduced the molten polymer’s flow resistance, providing good moldability for very thin and thicker parts.

## 5. Conclusions

The principal objective of the present review was to address the materials used in the coating of the mold cavity, but, concomitantly, they were able to have a positive impact on the cooling time during the injection molding cycle. The main conclusion is that these two different approaches are, in fact, distinct. Researchers working in optimizing the cooling channels, either by conventional or more recent technologies, are not concerned about the wear and corrosion problems in the mold cavity and vice-versa. The present review can be considered an alert to this lack of global perception in the injection molding industry and lead researchers to work together in solutions that can increase productivity and decrease rejected parts while also increasing the molds’ lifetime.

## Figures and Tables

**Table 1:** A brief summary of the research regarding the cooling system design of the injection mold process.

Reference and Year		Research Outcomes
[37]	2021	The design, manufacturing, and applications of conformational cooling channels are reviewed and evaluated systematically and comprehensively in this review paper.
[38]	2020	This survey focuses on the physical model, development, and optimization of conformal cooling systems that have curved cooling circuits following the shape of the mold cavity. Compared with traditional cooling systems, conformal cooling can greatly reduce the warpage defect and shorten the cooling cycle time.
[18]	2020	Experiments were performed with a plastic injection mold to confront and validate the simulations. Given the comparison of different cooling geometries, the simulations made it possible to obtain parts with shorter mold cooling cycles.
[39]	2019	Applying a constructal approach, the objective of the present work is to explore the thermal and hydraulic performances of radiant panels equipped with different flow architectures.
[40]	2019	The analysis and comparison of proposed channels show the advantage over conventional channels commonly used in injection molds. Results lead to the possibility of reducing the cooling phase and thus the production time of the entire injection cycle.
[9]	2019	It was possible to simulate injection processes in geometrically complex industrial molds, including those produced by additive manufacturing. These methods could be used to optimize the position and shape of the cooling channels in injection molding.
[1]	2018	The 3D results show the optimal configuration for the cooling channels for a complex injection chamber. This configuration allows minimizing the temperature in the solid domain guaranteeing structural resistance.
[41]	2018	Two different models allowed the quick determination of the cooling/solidifying time, the mold surface temperature variation, and the heat flux densities exchanged between the polymer and the mold. The good agreement between them validated these models’ interest in quickly getting reliable characteristic parameters of injection molding.
[7]	2017	The simulation results showed that the various sub groove designs give different values to ejection time. The addition of the sub groove significantly increased the coolant velocity and the rate of heat transfer from molten plastic to the coolant.
[8]	2017	Analysis of virtual models showed that those with conformal cooling channels predicted a significantly reduced cycle time and a marked improvement in the general quality of the surface finish compared to a conventionally cooled mold.
[33]	2015	The project of conformal cooling channels is very important. If the conformal cooling is not adequately designed, it cannot provide good results.
[19]	2014	The baseline design had an overall cooling surface area of 1.549.10^−5^ m^2^—was successfully managed to increase the area to 2.026.10^−5^ m^2^, a change of 30.8%, with the number of cooling channels increasing from five to seven.
[22]	2013	The thermal field in the mold is analyzed, and optimal regulation surfaces are extracted according to the shapes of some isotherms located in the quasi-stationary thermal zone of the mold. This led to the transition from a continuous distribution of the coolant fluid temperature to a discrete distribution of the cooling channels.
[42]	2010	The results indicate that, for the same cross-sectional area and coolant flow rate of the cooling channels, the form rectangular channels perform the minimum time required to solidify the plastic product completely.
[23]	2005	Analysis of virtual models showed that those with conformal cooling channels predicted a significantly reduced cycle time and a marked improvement in the general quality of the surface finish compared to a conventionally cooled mold.

**Table 2:** Comparison of the productivity of different cooling system geometries [44].

Cooling System	Total of Cyles (s)	Parts/Hour	Parts/Day	Parts/Year
“U”	22.0	1300	31,200	487,500
“Z”	21.9	1315	31,560	493,000
Rectangular	20.7	1390	33,360	521,250
Helical	19.5	1475	35,400	533,000

**Table 3:** Cooling system patents for injection molding, filed in the last decade.

Ref.	Year of Register	Register Number	Description
[46]	2015	US9089998 B2	Injection mold with a simplified cooling system. This patent proposes a simplified cooling channel system for molding at constant low-pressure injection. The cooling system is made with materials with high thermal conductivity, and when compared, by computer simulation, to other cooling systems with materials with both high thermal conductivity and high hardness, the simplified cooling system proved to be much more efficient.
[47]	2013	US20130295219A1	Injection mold having a simplified evaporative cooling system or a simplified evaporative cooling system with exotic cooling fluid. Evaporative cooling system. The plates with the channels have a higher thermal conductivity than the plates that form a molding cavity. When fluid is fluid in the channels, it evaporates, extracting heat from the plate with the cavity and condensation. The evaporative system has a condenser and a spray bar.
[48]	2012	US8105529B1	Heated injection molding system and method. A system and method are provided for molding a solid plastic part utilizing a mold having at least a core portion and a cavity portion which define a mold cavity. A pressurized fluid is introduced through a port on the mold core portion to bias the part being formed into engagement with the mold cavity. With the mold closed, pressurized steam is circulated through the mold cavity to heat the interior surface of the mold cavity. Once the mold cavity surface has reached the desired temperature, the pressurized steam is vented. When the condensate has been sufficiently removed, liquid plastic is injected into the mold to the mold cavity.

**Table 4:** A brief summary of the research results concerning the use of ceramic coating as a thermal barrier.

Reference and Year		Research Outcomes
[52]	2017	The simulation results for mold modified by yttria-stabilized zirconia (YSZ) powder used to coat an iron-based substrate exhibit a more homogeneous temperature profile. Employing the varying thickness thermal barrier coating influenced the injection molding process positively by helping to achieve a more uniform temperature distribution on the surface of the mold cavity, thus promising a reduction in warpage of plastic parts.
[25]	2015	This paper speculated about the possibility that DLC can act as a heat transfer buffer, weakening the influence of the heat transfer mechanism on the polymer/mold interface at the flow stage, allowing a less aggressive design of the temperature control system.
[26]	2015	The CVD of zirconia is a promising way to produce well adhering, uniform, and thermally isolating polished steel layers. Thick zirconium dioxide thermal barrier layers of up to 38 µm thick were successfully deposited, showing a reduced cooling rate, which is interesting in injection molding to avoid external induction heating and significantly reduce energy costs and cycling times.

## Data Availability

Not applicable.
